# Association of Immediate Release of Test Results to Patients With Implications for Clinical Workflow

**DOI:** 10.1001/jamanetworkopen.2021.29553

**Published:** 2021-10-18

**Authors:** Bryan D. Steitz, Lina Sulieman, Adam Wright, Samuel Trent Rosenbloom

**Affiliations:** 1 Department of Biomedical Informatics, Vanderbilt University Medical Center, Nashville, Tennessee; 2 Department of Medicine, Vanderbilt University Medical Center, Nashville, Tennessee; 3 Department of Pediatrics, Vanderbilt University Medical Center, Nashville, Tennessee

## Abstract

This cross-sectional study assesses changes in the volume of patient-initiated messages to clinicians associated with release of test results before and after implementation of the 21st Century Cures Act.

## Introduction

The 21st Century Cures Act (hereafter the Cures Act) mandates the immediate electronic release of all test results, medication lists, and clinical notes to patients without delay unless a rare allowable exception exists.^1^ Before implementation of the Cures Act, many institutions released only a subset of electronic health record (EHR) information through the patient portal. This information was often suppressed or delayed when it was considered by the health system to be highly sensitive or there was a risk of misinterpretation. The requirement that all electronic health information be immediately available to patients—who may see it before their clinicians—has raised concerns about unintended implications on clinical workflow and patient well-being.^2^ We examined the association of immediate release of test results with the percentage of results viewed first by patients as well as the number of messages that patients sent to their clinicians.

## Methods

This cross-sectional study included all health results that were released to the patient portal between January 1, 2020, and April 16, 2021. We performed this analysis at Vanderbilt University Medical Center (VUMC), which historically released all testing results to its patient portal, My Health At Vanderbilt (MHAV), based on tiered intervals according to test sensitivity and complexity.^3^ Delays in release of results to MHAV were calculated from the time when a test became available to physicians and other health care providers in the EHR. Cures Act compliance at VUMC began on January 20, 2021, by removing all information blocks that delayed test results from being shared to MHAV for adult patients. This study was approved by the Vanderbilt University Institutional Review Board, which granted a waiver of patient informed consent because the research was deemed less than minimal risk and the research was conducted on data previously collected during routine patient care. We followed the Strengthening the Reporting of Observational Studies in Epidemiology (STROBE) reporting guideline.

We measured the rates at which patients viewed their test results in the patient portal before their clinician reviewed them during the period that VUMC transitioned to Cures Act compliance. Measured outcomes included the percentage of tests seen by patients in MHAV before being seen by clinicians in the EHR and were stratified by the historic release delay categories: immediate release, or held for release after a 1-day, 3-day, 7-day, or 14-day delay. We also compared the median number of patient-initiated messages sent to clinicians within 6 hours of reviewing a result before and after the transition to Cures Act compliance. Although data on race and ethnicity are collected as part of routine care and were contained within the EHR, analyzing these data would require additional consideration of the implications and was beyond the scope of this analysis. All analyses were conducted in R, version 4.0.2 (R Foundation for Statistical Computing).

## Results

Between January 1, 2020, and April 16, 2021, 294 799 patients (mean [SD] age at Cures Act compliance, 47.3 [17.8] years; 184 711 women [62.7%] and 110 088 men [37.3%]) received 3 221 394 test results in MHAV. Among these patients, there were 265 923 (90.2%) who reviewed 2 661 441 (82.6%) results. The Figure shows the percentage of results over time that were reviewed first by patients. Among tests categorized to be released after any delay before January 20, 2021, patients viewed 10.4% of results before clinicians compared with 40.3% of the same results after January 20, 2021. The number of daily messages sent by patients within 6 hours of reviewing a test released after any delay changed from a median [IQR] of 77.5 [13.75-105.25] messages before the transition to Cures Act compliance to 146 [12.0-169.0] messages after the transition.

**Figure.**
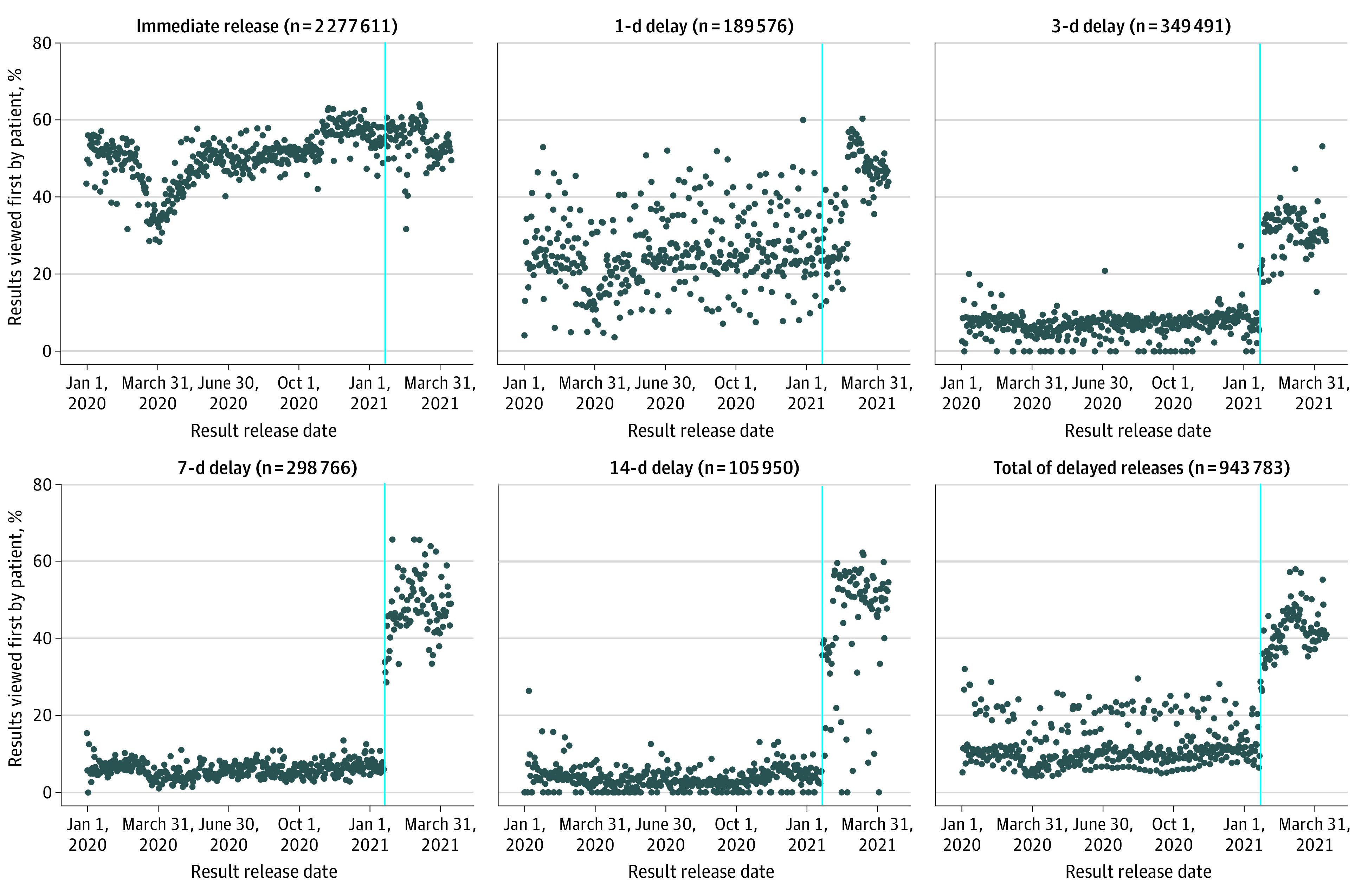
Results Reviewed First by Patients Stratified by Immediate or Delayed Release The blue lines represent January 20, 2021, the date when Vanderbilt University Medical Center began compliance with the 21st Century Cures Act.

## Discussion

Improved availability of data to patients represents a marked transformation in patients’ opportunity to take ownership of their health care.^2^ However, the benefit associated with immediate release of test results may be overshadowed by unintended consequences to patient well-being and clinical workload.^2^ Additional consideration of the timing of test result release to patients and clinicians is necessary to ensure that results are made available to patients while maintaining the opportunity for clinicians to apply their expertise and interpretation.

This study has limitations. First, the study was conducted at a single academic medical center, limiting the generalizability of the findings. Second, we measured changes in the number of patient-initiated messages relative to the timing of result review without further analysis of message content. Additional research is necessary to assess patient and physician perceptions of reviewing immediately released test results, which warrants future research.
